# Light enough to travel: migratory bats have smaller brains, but not larger hippocampi, than sedentary species

**DOI:** 10.1098/rsbl.2010.0744

**Published:** 2010-09-29

**Authors:** Liam P. McGuire, John M. Ratcliffe

**Affiliations:** 1 Department of Biology, University of Western Ontario, London, Ontario, CanadaN6A 5B7; 2 Institute of Biology, University of Southern Denmark, 5230 Odense M, Denmark

**Keywords:** bats, brain size, hippocampus, migration, neocortex

## Abstract

Migratory bird species have smaller brains than non-migratory species. The behavioural flexibility/migratory precursor hypothesis suggests that sedentary birds have larger brains to allow the behavioural flexibility required in a seasonally variable habitat. The energy trade-off hypothesis proposes that brains are heavy, energetically expensive and therefore, incompatible with migration. Here, we compared relative brain, neocortex and hippocampus volume between migratory and sedentary bats at the species-level and using phylogenetically independent contrasts. We found that migratory bats had relatively smaller brains and neocortices than sedentary species. Our results support the energy trade-off hypothesis because bats do not exhibit the same degree of flexibility in diet selection as sedentary birds. Our results also suggest that bat brain size differences are subtler than those found in birds, perhaps owing to bats' shorter migration distances. Conversely, we found no difference in relative hippocampus volume between migratory and sedentary species, underscoring our limited understanding of the role of the hippocampus in bats.

## 1. Introduction

Several studies have examined avian brain size in relation to migratory behaviour at the species [1–3] and sub-species level [4,5]. Two leading hypotheses to explain observed differences are the behavioural flexibility/migratory precursor hypothesis [1] and the energy trade-off hypothesis [6]. The behavioural flexibility hypothesis suggests that sedentary species face changing environmental conditions over the course of the year and therefore, must be flexible in foraging behaviour and dietary breadth. It follows that because larger brains confer greater behavioural flexibility they are selected for in sedentary species, while species with relatively smaller brains are not capable of such flexibility and instead migrate to remain within favourable habitat.

Conversely, the energy trade-off hypothesis argues that because the brain is an energetically expensive organ to maintain [7], animals should partially offset its costs through minimizing others, including the costs of locomotion. Large brains may be particularly problematic for flying organisms because increased mass contributes substantially to the energetic costs of flight [8,9]. The energy required for migration may limit that available for brains and, inversely, larger brains may increase migration costs. Migratory species are thus expected to have smaller brains than sedentary species [3,6].

Studies have also considered variation in avian hippocampus size (e.g. [10]) and mammalian neocortex size (e.g. [11]). The hippocampus is involved in spatial memory in birds and mammals and hence may be important in bats for recalling landmarks and migratory routes. Although the role of the hippocampus is poorly understood in bats, we might expect to observe patterns similar to those in birds, where migratory species have relatively larger hippocampi than sedentary species [10]. In mammals, relative neocortex size correlates with enhanced cognition [11,12]. In predatory bats, its relative size is reduced in bats that aerially hawk prey in open spaces, species that tend to have larger home ranges than other predatory bats and wings better suited to long-distance flight [13,14].

Addressing the issue of brain size in relation to migration in non-avian vertebrates could reveal general patterns of brain size evolution [1]. Bats and birds are the only extant vertebrate groups capable of powered flight and so may be subject to similar selective pressures [15]. Like birds, some bats undertake seasonal migrations and thus experience similar environmental conditions throughout the year [16]. However, few sedentary bats exhibit behavioural flexibility with respect to seasonal diet change on par with that which has been observed in many sedentary birds [15].

Furthermore, in temperate zones sedentary birds often experience dramatic environmental variation, while many species of bat (migratory and sedentary) simply hibernate. As a result, in many regions both sedentary and migratory bats that hibernate experience limited seasonal variation. Thus, neither sedentary nor migratory bat species should require as great a degree of overall behavioural flexibility as do most sedentary birds. We therefore posit that if migratory bats have relatively smaller brains than sedentary species this result would better support the energy trade-off hypothesis than the behavioural flexibility hypothesis in flying vertebrates.

## 2. Material and methods

### (a) Data assembly

**Figure 1.**
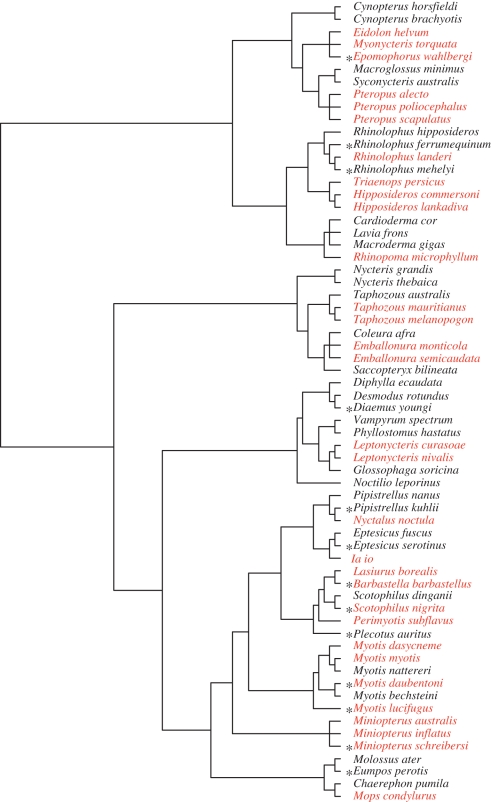
Composite phylogeny used to generate phylogenetically independent contrasts (PICs). Migratory species are indicated in red, sedentary species in black. Species marked with an asterisk had only whole brain, not brain region. Data are available in the electronic supplementary material S1. For details of phylogeny construction see electronic supplementary material S2.

Baron *et al*. [17] includes brain and body mass data for 342 bat species and specific brain region data for a subset of these species. We confined ourselves to these species and searched the literature for those that had been documented to have a migratory or a sedentary lifestyle (figure 1 and electronic supplementary material, S1 for supporting references). Because bats are mostly small and nocturnal, bat migration has been historically understudied; our list of species is almost certainly an underestimate. We also found sedentary species difficult to identify because staying put is the rule in bats and thus rarely explicitly described. To supplement our list of species explicitly described as sedentary, we included those for which there is no mention of migration and year-round, population-specific reproduction at the same location has been documented (figure 1 and electronic supplementary material S1).

### (b) Comparative analyses

We took the standardized residuals from log–log regressions of brain volume (converted from brain mass, see electronic supplementary material S1) versus body mass, and neocortex and hippocampus volume versus brain volume remainder. Additionally, we used medulla oblongata and cerebellum volume versus brain volume remainder as controls. The cerebellum's primary function is motor control and calibration; the medulla oblongata (i.e. the lower portion of the brainstem) controls several autonomic functions (e.g. heart rate and respiration). Neither region was expected to differ with migratory status (electronic supplementary material S1). For analyses at the species-level (SL) and to generate phylogenetically independent contrasts (PICs), we used these standardized residuals as data. We conducted two-sample *t*-tests at the SL. For those using PICs (generated by Brunch procedure in CAIC v. 2.6.9), we conducted one-sample *t*-tests [18]. For CAIC analyses, we constructed a composite phylogeny (figure 1; see electronic supplementary material S2 for details of phylogeny construction). At both levels of analysis, all tests were two-tailed.

## 3. Results

Log brain volume was positively related to log body mass (*F*_1,62_ = 774.2, *r*^2^ = 0.93, *p* < 0.001), as were log brain region volumes to their respective log brain volume remainders (hippocampus: *F*_1,50_ = 605, *r*^2^ = 0.92, *p* < 0.001; neocortex: *F*_1,50_ = 1835.2, *r*^2^ = 0.97, *p* < 0.001; medulla oblongata: *F*_1,50_ = 1085.2, *r*^2^ = 0.96, *p* < 0.001; cerebellum: *F*_1,50_ = 770.5, *r*^2^ = 0.94, *p* < 0.001).

Absolute and log-transformed body mass values did not differ significantly between migratory and sedentary species (two two-sample *t*-tests: *p* > 0.05 for both; electronic supplementary material S1).

**Figure 2.**
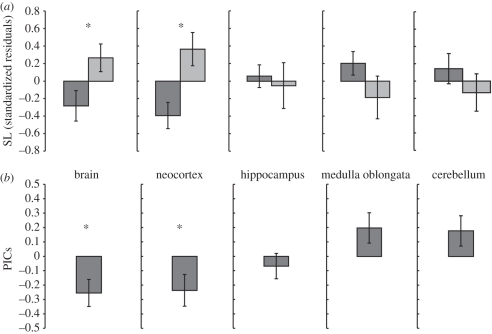
(*a*) At the SL, five two-sample *t*-tests comparing relative brain and brain region volumes (transformed as described in §2) in migratory bats to those of sedentary bats. (*b*) Based on PICs (generated as described in §2), five one-sample *t*-tests comparing mean relative brain and region volumes in migratory bats to those for all species pooled (combined mean set to 0 for all). Data are presented as mean ± s.e. (asterisk indicates *p* < 0.05). Dark grey boxes, migratory; light grey boxes, sedentary.

At the SL and for PICs, relative brain size was significantly greater in sedentary species than in migratory species (SL: *t* = 2.26, *p* = 0.027; PICs: *F*_1,16_ = 7.27, *p* = 0.016; figure 2). Similarly, at both levels of analysis, relative neocortex size was significantly greater in sedentary species (SL: *t* = 2.94, *p* < 0.006; PICs: *F*_1,14_ = 4.6, *p* = 0.049; figure 2). Relative hippocampus (SL: *t* = −0.39, *p* = 0.7; PICs: *F*_1,14_ = 0.59, *p* = 0.453), medulla oblongata (SL: *t* = −1.42, *p* = 0.16; PICs: *F*_1,14_ = 3.48, *p* = 0.083) and cerebellum (SL: *t* = −0.99, *p* = 0.325; PICs: *F*_1,14_ = 2.83, *p* = 0.11) volumes did not differ between categories (figure 2).

## 4. Discussion

Our comparative analyses suggest that migrating bats have relatively smaller brains and neocortices than sedentary species (figure 2) but do not differ significantly in body mass, supporting the energy trade-off hypothesis. In both bats and birds, two distantly related vertebrate groups with divergent life histories, migratory species have relatively smaller brains than do sedentary species, suggesting a general incompatibility between the high energy demands of migration and those of maintaining and carrying a large brain. In bats, however, the effect appears not to be as profound as in birds. In some bird species, brain size is negatively related to migration distance [3]. Few bat species are, relative to birds, long-distance migrants and this discrepancy may account, in part, for the apparently smaller effect size in bats.

By contrast, we found no difference in relative hippocampus size between migratory and sedentary bats (figure 2). In birds, an enlarged hippocampus has been linked to migration [10], but also to smaller scale spatial memory [19]. While the role of the hippocampus in bats remains unclear, it may function in migratory navigation [20] and, in frugivorous and nectarivorous species, for the relocation of food [21]. Confoundingly, among phyllostomids, gleaners have relatively larger hippocampi than even frugivores and nectarivores [22]. Currently, limited data and the potential for multiple roles preclude making clear predictions about hippocampus size and bat migration.

Brain and brain region size variation in bats has been considered in relation to several behavioural, physiological and ecological factors (see [12] for review). While a variety of factors will influence observed phenotype, our results suggest that energetic limitations play a major role in determining brain size in migrating bats. Further support for the energy trade-off hypothesis comes from studies of brain size and foraging strategies in bats. Brain and neocortex size is smallest in obligate aerially hawking bats foraging primarily in open spaces [13,14], consistent with the idea that the energetic requirements of high-powered fast flight (open space aerial-hawking and/or migratory flight) negatively impact brain size. Whether a bat species' relative brain and brain region size reflects a migratory or sedentary evolutionary history, as our data suggest, or waxes and wanes as a result of individual experience, as in some migratory birds (e.g. [2,19]), are among a number of possibilities. Indeed, research into migration and brain development in birds has revealed many unexpected and puzzling factors (e.g. species-specific, latitudinal effects, basal metabolic rate, diet, developmental constraints) and we caution that ours is a preliminary study, demonstrating a correlative, not causal, relationship (see Dechmann & Safi [12] for review). Furthermore, it should be noted that migration is a characteristic of individuals, not species. Differential and partial migration are both common among bats with many examples of sex-biased migration, and migratory and non-migratory populations within species. Whatever the underlying and interacting selective forces, future studies comparing bat and birds should yield further insight into the processes of vertebrate brain evolution.
